# Knowledge, perceptions, and experiences of family caregivers and home care providers of physical restraint use with home-dwelling elders: a cross-sectional study in Japan

**DOI:** 10.1186/1471-2318-14-39

**Published:** 2014-03-27

**Authors:** Sadami Kurata, Toshiyuki Ojima

**Affiliations:** 1 Gerontological Nursing, Faculty of Nursing, Hamamatsu University School of Medicine, Hamamatsu, Japan; 2 Department of Community Health and Preventive Medicine, Hamamatsu University School of Medicine, Hamamatsu, Japan

**Keywords:** Physical restraints, Aged, Family caregivers, Home care providers

## Abstract

**Background:**

The use of physical restraints by family caregivers with home-dwelling elders has not been extensively studied but it might be widespread. Furthermore, it is also not clear how home care providers who support family caregivers perceive the use of physical restraint in elders’ homes. This study assessed family caregivers’ and home care providers’ knowledge and perceptions of physical restraint used with elders living at home in Japan, a country with the highest proportion of elders in the world and where family caregiving is common.

**Methods:**

We undertook a cross-sectional study of 494 family caregivers, 201 home helpers, 78 visiting nurses, 131 visiting physicians, and 158 care managers of home-dwelling frail elders needing some care and medical support in Japan, using questionnaires on knowledge of 11 physical restraint procedures prohibited in institutions and 10 harmful effects of physical restraints, perceptions of 17 reasons for requiring physical restraints, and experiences involving physical restraint use.

**Results:**

Family caregivers were aware of significantly fewer recognized prohibited physical restraint procedures and recognized harmful effects of physical restraint than home care providers, and differences among home care providers were significant. The average importance rating from 1 (*least*) to 5 (*most*) of the 17 reasons for requiring physical restraints was significantly higher among family caregivers than home care providers, and significantly different among the home care providers. Moreover, these differences depended in part on participation in physical restraint education classes. While 20.1% of family caregivers had wavered over using physical restraints, 40.5% of home care providers had seen physical restraints used in elders’ homes and 16.7% had advised physical restraint use or used physical restraints themselves.

**Conclusions:**

Knowledge and perceptions of physical restraints differed between family caregivers and home care providers and were also diverse among home care providers. Because both groups might be involved in physical restraint use with home-dwelling elders, home care providers should acquire standardized and appropriate knowledge and perceptions of physical restraints to help family caregivers minimize abusive physical restraint use.

## Background

Physical restraints might be used frequently with not only institutionalized elders [1] but also home-dwelling elders. It has been suggested, for example, that physical restraints might be commonly used in home care in the Netherlands [2,3] and that dementia patients might be restrained by family caregivers as a form of abuse or neglect in the United States [4]. In Japan, the preliminary results of a study reported that 19.4% of family caregivers perceived the need for physical restraints with home-dwelling elders [5], and 423 of 916 expert advisors on assistive products had been asked by family caregivers for assistive devices to provide physical restraint [6]. However, the actual state of physical restraint use by family caregivers has not been extensively studied [3,4]. One study has investigated physical restraint use by nursing staff in home care settings [2], and several studies have examined how care providers such as nurses perceive the issue of physical restraint use in nursing homes and hospitals and how families perceive the use with institutionalized elders [7-10]. However, no studies have investigated how home care providers who support family caregivers perceive physical restraints used in elders’ homes.

Japan has the highest proportion of people aged ≥65 years in the world, just as traditional support for elderly people is declining [11]. In response to this, Japan’s public mandatory long-term care insurance (LTCI) was implemented in 2000 to support the independent routines of elders in daily life and relieve the burden on family caregivers [11]. As a result, frail elders have access to many LTCI services if they meet LTCI eligibility criteria based on daily life activities. For home-dwelling elders, a care manager arranges services, which include home helpers (housekeeping and personal care), visiting nurses, day care, day care with rehabilitation, and short-stay respite care [12,13]. The main providers of in-home care for the elderly in Japan are care managers, home helpers, and visiting nurses, all of whom are well acquainted with the daily life situation of the care recipients. Care managers come from related occupations, including care workers, nurses, physicians, pharmacists, and are certificated by passing an examination and completing a 44-hour training course. Nearly all care recipients choose to have a care manager who will draw up a care plan for home care services based on LTCI and will coordinate with other home care providers [11]. Home helpers are certificated mostly by completing at least 130 hours of theoretical education and practical training. Visiting nurses are nationally certified registered nurses or assistant nurses who are licensed by a prefectural governor. Most visiting physicians are family physicians whose only qualification in Japan is a physician’s license, and they provide in-home medical management in cooperation with visiting nurses.

In the first 10 years of operation, Japan’s LTCI program has seen a 2.2-fold increase in the number of users, with 2.18 million and 4.87 million users in 2000 and 2010, respectively [14]. Similarly, the number of elders with dementia has doubled from 1.49 million in 2001 to 3.05 million in 2012, and that of elders with the greatest care needs has been increasing rapidly [15]. Among all LTCI users, 22% are LTCI facility residents [16], which corresponds to only 4% of people aged ≥65 years in Japan. The proportion of elders living with any child is still 42.2%, and most live at home and receive family care [17,18]. In other Asian countries, the proportion is also high [19]. Even in the United States, the number of multi-generational family households is increasing and family caregiving of disabled elders has become commonplace; for example, among adults aged ≥65 years, the proportion of those living in multi-generational households increased from 16.8% in 1990 to 19.6% in 2008 [20]. Family caregiving is thus common in most countries.

Family caregiving for the elderly may, however, involve using physical restraints and thus have the potential for elder abuse through abusive physical restraint use [4,21]. Before LTCI implementation in Japan, numerous people aged ≥65 years were living in hospitals with little medical justification [11], and physical restraints were commonly used in hospitals to prevent accidents such as falling out of bed and pulling out medical tubes [22]. However, Japan’s LTCI policy deemed physical restraint use a violation of human rights, and therefore legally prohibited physical restraint procedures in LTCI facilities [23]. After this prohibition, physical restraint use decreased by 47% in nursing homes (i.e., facilities covered by public aid providing long-term care to the elderly) and by 35% in homes providing more medical care (i.e., long-term care health facilities) [24]. In contrast, in a recent national survey in Japan, the number of incidents of elder abuse was found to have increased from 12,623 in 2006 to 16,750 in 2011, with the highest proportion of cases involving physical abuse including physical restraint use, and 99% of cases of elder abuse occurred at home, of which 86% were perpetrated by the family [25]. The abuse of home-dwelling elders might then have much to do with unnecessary and abusive physical restraint use by family caregivers.

Unlike in institutionalized settings, the issue of physical restraint use by family caregivers in home care settings might be too sensitive for a direct survey of the prevalence of family caregivers’ use, at least in Japan. Therefore, as an initial approach to reducing unnecessary and abusive physical restraint use by Japanese family caregivers, in this study we assessed family caregivers’ and home care providers’ knowledge and perceptions of physical restraint use with home-dwelling elders to determine whether the actual conditions behind physical restraint use by family caregivers needs to be investigated in detail. Based on our clinical experience, we hypothesized that the degree of knowledge and perceptions of physical restraint use might differ between family caregivers and home care providers and may vary among home care providers themselves, which could detrimentally affect interventions designed to reduce family caregiver’s physical restraint use. We also included in the survey items regarding the physical restraints used in home care and education received about physical restraints, as such data might help to determine the need to survey in more detail the prevalence of physical restraint use by family caregivers and the importance of education on physical restraint use.

## Methods

### Study design and setting

This cross-sectional study was conducted in Hamamatsu and Iwata cities in Western Shizuoka prefecture located in central Japan, locations we selected based on convenience sampling. From 196 visiting in-home care management offices, 100 home helper stations, and 35 visiting nurse stations in the two cities, we selected purposive and volunteer samples; that is, we sampled those that had provided services for at least several years and had accepted by telephone our request to visit. We also sampled all 294 medical clinics that provided home visiting by physicians in the two cities. Data collection, including visiting and mailing, took place between September 2007 and March 2009. The study was approved by the Ethics Review Board of Hamamatsu University School of Medicine (No. 19–6).

### Participants and recruitment procedures

The participants consisted of family caregivers and home care providers (home helpers, visiting nurses, visiting physicians, and care managers) who provide caregiving to home-dwelling elders. To recruit family caregivers and home care providers other than visiting physicians, we visited 79 in-home care management offices, 31 home helper stations, and 15 visiting nurse stations, which were sampled as described above. We mailed invitations to participate to all 294 relevant medical clinics to recruit visiting physicians.

**Figure 1:**
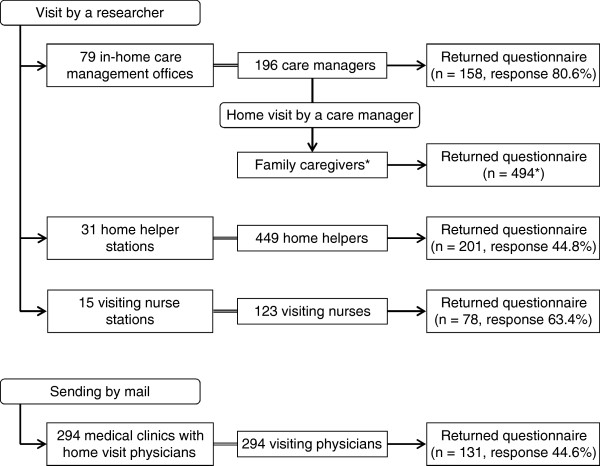
**Flow chart of participant recruitment with response rates.** *We did not require care managers to report the number of family caregivers they had approached. Therefore, the total number approached and the response rate are unknown.

Family caregivers of frail elders living at home with family but who still needed some care and medical support were recruited with the assistance of 196 care managers at 79 in-home care management offices (multistage sampling; Figure 1). First, a researcher visited the agency head of each office to explain the study’s objectives. Second, each agency head explained the objectives to the office’s care managers. Third, care managers who agreed to participate explained the objectives to all eligible family caregivers during routine home visits. Finally, questionnaires (enclosed in envelopes) were given by care managers to the family caregivers to complete.

We also applied multistage sampling (Figure 1) to recruit home care providers. First, a researcher visited 31 home helper stations, 15 visiting nurse stations, and 79 in-home care management offices to explain the study’s objectives to the head of each station or office. Then, the agency head handed out questionnaires for home care providers to 449 home helpers, 123 visiting nurses, and 196 care managers. For visiting physicians, we mailed questionnaires for home care providers to all 294 clinics. Participants were deemed to have given informed consent by returning the anonymous questionnaires directly by mail to one of the researchers.

### Measurements

We asked all participants to complete anonymous self-administered questionnaires on (1) knowledge of 11 physical restraint procedures prohibited by LTCI facilities and 10 harmful effects of physical restraints, (2) perceptions of 17 reasons for requiring physical restraints; and (3) experiences involving physical restraint use. The primary and secondary outcome measures were knowledge and perceptions of physical restraint use and experiences of its use, respectively. Questionnaires (1) and (2) corresponded to the primary outcome measure and questionnaire (3) to the secondary outcome measure. As background variables, we asked for participants’ characteristics, including sex and age (by decade), and for family caregivers only, about their relationship to the elders they cared for and the duration of caregiving.

### Knowledge of prohibited physical restraints and their harmful effects

In the Guidebook for Zero Physical Restraint Campaign produced by the Japanese Ministry of Health, Labour and Welfare [26], 11 procedures (Table 1) are specifically indicated as physical restraints prohibited in care facilities. The knowledge of each physical restraint procedure was assessed by the following question: “Do you think the procedure is one of the physical restraints prohibited in principle?” Response options were “yes,” “no,” and “not sure.” Reliability as assessed by Cronbach’s alpha was 0.84. The Guidebook also lists 10 harmful effects of physical restraints (Table 1). The recognition of each harmful effect was assessed by the following question: “Do you think physical restraints may cause harmful effects?” Response options were “yes,” “no,” and “not sure.” Reliability as assessed by Cronbach’s alpha was 0.85.

**Table 1:** Prohibited physical restraints, harmful effects of physical restraints, and reasons requiring physical restraints


**11 Physical restraint procedures prohibited in facilities**	
P1	Tying a person to a wheelchair/bed to prevent wandering
P2	Tying a person to a bed for fall prevention
P3	Using siderails to keep a person in bed
P4	Tying limbs to prevent a person from pulling out IV/feeding tubes
P5	Applying mittens to prevent a person from pulling out IV/feeding tubes or tearing skin
P6	Restricting a person with belts or tray tables to prevent sliding or rising from a (wheel)chair
P7	Using a chair to prevent a person from being able to stand up
P8	Using overalls over clothing to impede removal of clothes/diapers
P9	Tying a person to a bed to prevent them from causing trouble to others
P10	Giving an overdose of psychotropic drugs to reduce excitement
P11	Locking a person in a room
**10 Harmful effects of physical restraints**	
H1	Muscle weakness, joint contracture, pressure ulcer
H2	Reduced appetite, cardiopulmonary function, or immunity
H3	Falling by standing up from a wheelchair fixed with a device
H4	Falling down by climbing over bed rails
H5	Suffocation by restraining devices
H6	Anxiety and aggression, feelings of shame, disillusionment, loss of motivation, and self-respect
H7	Progression of dementia and becoming more susceptible to delirium
H8	Family feelings of regret and guilt
H9	Further medical treatment and economic burden
H10	Vicious cycle of physical restraints as a result of declining strength, worsening dementia, delirium, and physical restraint-induced falls
**17 Reasons for requiring physical restraints**	
R1a	Protecting an older person from falling out of bed
R1b	Protecting an older person from falling out of a chair
R1c	Protecting an older person from falling due to unsafe ambulation
R2	Preventing an older person from wandering
R3	Preventing an older person from taking things from others
R4	Preventing an older person from getting into dangerous places or supplies
R5	Keeping a confused older person from bothering others
R6a	Preventing an older person from pulling out a catheter
R6b	Preventing an older person from pulling out a feeding tube
R6c	Preventing an older person from pulling out an intravenous tube
R6d	Preventing an older person from breaking open sutures
R6e	Preventing an older person from removing a dressing
R7	Providing quiet time or rest for an overactive older person
R8	Providing for safety when judgment is impaired
R9	Insufficient staff to observe patients
R10	Protecting staff or other patients from physical abuse/combativeness
R11	Management of agitation

### Perceived reasons for requiring physical restraints

We evaluated perceived reasons for requiring physical restraints by administering the Japanese version of the Physical Restraint Use Questionnaire [27-29]. For each of 17 reasons why elders might be physically restrained (Table 1), participants were asked to rate the reason’s importance on a 5-point Likert scale (1 = least important, 5 = most important): the higher the average score, the more favorable the respondent’s overall perception of using physical restraints with elders. Reliability as assessed by Cronbach’s alpha was 0.94.

### Experiences with and education classes about physical restraints

To assess family caregivers’ experiences involving physical restraint use, we asked whether they had ever wavered over using physical restraints with elders for whom they cared (response options: “yes” or “no”) and who they would consult to resolve their uncertainty about using such restraints (response options: “other family members,” “home helpers,” “visiting nurses,” “visiting physicians,” “care managers,” “staff of day care centers,” or “others”). To assess home care providers’ experiences involving physical restraint use, we asked whether they had ever seen physical restraints used with home-dwelling elders and whether they had recommended using them or ever used them themselves in elders’ homes (response options: “yes” or “no”). In addition, we asked family caregivers and home care providers whether they had attended any education classes on physical restraints (response options: “none,” “once,” or “twice or more”).

### Statistical analysis

Statistical analysis was performed using SPSS Version 17.0 J for Windows (SPSS Inc., Chicago, IL). For normally distributed continuous data, we compared means between two groups using the unpaired *t*-test, and means among 3 or more groups using ANOVA followed by the Bonferroni test. To compare variables with a non-normal distribution, we used the Mann–Whitney *U* test or the Kruskal-Wallis test followed by the Mann–Whitney *U* test with Bonferroni adjustment. We used the chi-square test to compare categorical variables.

## Results

### Participant characteristics

Questionnaires were returned by 494 family caregivers (the response rate could not be calculated because the total number of family caregivers approached by the care managers was unknown), 201 home helpers (response rate: 44.8%), 78 visiting nurses (63.4%), 131 visiting physicians (44.6%), and 158 care managers (80.6%). Age and sex were significantly different between family caregivers, helpers, nurses, physicians, and care managers (both p < .001; Table 2). The mode of age was 50 years for family caregivers, helpers, and physicians and 40 years for nurses and care managers. Sex was predominantly female among helpers, nurses, care managers, and family caregivers and predominantly male among physicians. The relationship of the family caregiver to the elder cared for was daughter-in-law for 125 participants (25.3%), daughter for 119 (24.1%), wife for 100 (20.2%), son for 59 (11.9%), husband for 37 (7.5%), and other for 32 (including no response for 22). Mean duration and standard deviation of care by family caregivers was 57.2 ± 52.6 months (range 1–336 months).

**Table 2:** Type, age (decade), and sex of participants

	**Family caregiver**	**Home care provider**	**Home helper**	**Visiting nurse**	**Visiting physician**	**Care manager**
	**n = 494**	**n = 568**	**n = 201**	**n = 78**	**n = 131**	**n = 158**
**Age, n (%)**						
20s	0 (0)	5 (0.9)	2 (1.0)	0 (0)	0 (0)	3 (1.9)
30s	17 (3.6)	99 (17.6)	28 (14.2)	27 (34.6)	4 (3.1)	40 (25.5)
40s	41 (8.6)	167 (29.7)	54 (27.4)	38 (48.7)	24 (18.5)	51 (32.5)
50s	157 (32.9)	204 (36.3)	85 (43.1)	12 (15.4)	58 (44.6)	49 (31.2)
60s	142 (29.8)	66 (11.7)	26 (13.2)	1 (1.3)	25 (19.2)	14 (8.9)
70s	88 (18.4)	21 (3.7)	2 (1.0)	0 (0)	19 (14.6)	0 (0)
≥80s	32 (6.7)	0 (0)	0 (0)	0 (0)	0 (0)	0 (0)
**Sex, n (%)**						
Female	367 (77.8)	358 (79.6)	164 (96.5)	60 (93.8)	13 (15.1)	121 (93.1)
Male	105 (22.2)	92 (20.4)	6 (3.5)	4 (6.3)	73 (84.9)	9 (6.9)

*Note.* Due to missing data, the sum of participants in age or sex subgroups within each group is smaller than the total number of those within each group.

### Knowledge of prohibited physical restraints

The questionnaire on physical restraint procedures prohibited in principle was completed by 490 family caregivers, 201 helpers, 78 nurses, 130 physicians, and 158 care managers. The number of physical restraint procedures recognized as prohibited physical restraints differed significantly between family caregivers and home care providers (p < .001; Table 3) and among the 4 subgroups of home care providers (i.e., helpers, nurses, physicians, and care managers) (p < .001). The number of procedures recognized was significantly higher among care managers than physicians, nurses, and helpers (all p < .001) and significantly lower among physicians than helpers (p = .03). For each of the 11 physical restraint procedures, the frequency of recognition of prohibited physical restraints differed significantly between family caregivers and home care providers (all p < .001) and among home care providers (all p < .001). For all participants (i.e., family caregivers and home care providers), the frequency was highest among care managers for all physical restraint procedures and lowest among family caregivers for all physical restraint procedures except for “applying mitten gloves to prevent removal of IV/feeding tubes or tearing skin,” which was the lowest among physicians (physicians 15.5%, family caregivers 19.0%, nurses 21.8%, helpers 27.4%, and care managers 43.9%). Additional file 1 shows the results for each physical restraint procedure.

**Table 3:** Numbers of recognized prohibited physical restraints and harmful effects, and importance ratings of reasons for requiring physical restraints

	**Mean ± SD (Median) (95% Confidence Interval of Mean)**		
	**Number of physical restraint procedures recognized as prohibited**	**Number of harmful effects recognized**	**Average importance rating of reasons for requiring physical restraints**
Total	5.33 ± 3.56 (5) (5.11-5.55)	7.02 ± 3.02 (8) (6.84-7.21)	2.94 ± 0.79 (3.00) (2.89-2.99)
*Participant groups*			
Family caregiver	3.86 ± 3.34 (3) (3.56-4.17)	5.88 ± 3.30 (6) (5.58-6.18)	3.21 ± 0.78 (3.25) (3.14-3.29)
Home care provider	6.57 ± 3.27 (7) (56.29-6.85)	8.00 ± 2.35 (9) (7.80-8.20)	2.72 ± 0.72 (2.75) (2.66-2.78)
Home helper	6.29 ± 3.32 (6) (5.81-6.76)	7.81 ± 2.50 (8.5) (7.45-8.16)	2.88 ± 0.69 (2.94) (2.78-2.98)
Visiting nurse	5.85 ± 3.21 (5) (5.11-6.59)	7.85 ± 2.23 (8) (7.34-8.35)	2.84 ± 0.62 (2.81) (2.69-2.98)
Visiting physician	5.19 ± 2.92 (5) (4.68-5.71)	7.20 ± 2.74 (8) (6.72-7.68)	2.74 ± 0.75 (2.81) (2.61-2.88)
Care manager	8.44 ± 2.65 (9) (8.01-8.86)	8.99 ± 1.36 (10) (8.78-9.21)	2.44 ± 0.70 (2.31) (2.32-2.55)
p-value^*^	< .001	< .001	< .001

SD = Standard Deviation.

^*^p-value for differences among 4 home care provider subgroups (home helpers, visiting nurses, visiting physicians, and care managers).

### Recognition of harmful effects of physical restraints

The questionnaire on harmful effects of physical restraints was completed by 491 family caregivers, 200 helpers, 78 nurses, 130 physicians, and 158 care managers. The number of recognized harmful effects differed significantly between family caregivers and home care providers (p < .001; Table 3) and among the 4 home care provider subgroups (p < .001). The number recognized was significantly higher among care managers than physicians (p < .001), helpers (p = .001), and nurses (p = .03). For each of the 10 harmful effects, the frequency of recognition differed significantly between family caregivers and home care providers (all p < .001) and among home care providers (all p < .001). For all participants, the frequency of recognition for all harmful effects was highest among care managers and was lowest among family caregivers except for “further medical treatment and economic burden,” which was lowest among physicians (physicians 36.9%, family caregivers 42.2%, nurses 48.7%, helpers 53.1%, and care managers 67.9%). Additional file 1 shows the results for each harmful effect of physical restraints.

### Perceived reasons for requiring physical restraints

The questionnaire on perceived reasons for requiring physical restraints was completed by 472 family caregivers, 197 helpers, 78 nurses, 130 physicians, and 158 care managers. The average importance rating (1 = least, 5 = most) of the 17 reasons for requiring physical restraints differed significantly between family caregivers and home care providers (p < .001; Table 3) and among the 4 home care provider subgroups (p < .001). The average rating was significantly lower among care-managers than helpers (p < .001), nurses (p = .002), and physicians (p = .007). For all participants, the average rating was highest among family caregivers for all reasons except for “protecting an older person from falling out of bed” (group means: care managers 2.61, helpers 3.08, nurses 3.13, family caregivers 3.16, and physicians 3.27) and “protecting an older person from falling out of a chair” (care managers 2.54, nurses 2.90, helpers 2.94, family caregivers 3.07, and physicians 3.13), and was lowest among care managers for all reasons except for “providing quiet time or rest for an overactive older person,” which was lowest among physicians (physicians 1.70, care managers 1.78, nurses 2.08, helpers 2.14, and family caregivers 2.54). Additional file 1 shows results of the importance ratings for each reason.

### Experiences involving physical restraints

Among 443 family caregivers who answered the question about personally using physical restraints, 89 (20.1%) had wavered over their use with home-dwelling elders. Among 285 family caregivers who answered the question about who they would consult when uncertain about using physical restraints, 164 (57.5%) chose care managers, 142 (49.8%) visiting physicians, 109 (38.2%) other family members, 59 (20.7%) staff of day care centers, 37 (13.0%) visiting nurses, 29 (10.2%) home helpers, and 8 (2.8%) others (total exceeds 100% due to multiple-choice answers).

Among 555 home care providers (response rate: 52.3%) who answered the question about observing physical restraint use in elders’ homes, 225 (40.5%) had seen it used. The proportion differed significantly among helpers, nurses, physicians, and care managers, with the proportion among nurses and care managers more than twice that among physicians (Table 4). Moreover, among 550 home care providers who answered the question about providing advice on physical restraint use or personally using physical restraints in homes, 92 (16.7%) had done so. The proportion differed significantly among helpers, nurses, physicians, and care managers, with the proportion among nurses more than twice as high as that among physicians and helpers (Table 4).

**Table 4:** Comparisons among home care providers’ experiences regarding physical restraint use and participation in education classes

	**Home helper**	**Visiting nurse**	**Visiting physician**	**Care manager**	**Total**	**p-value**
*Have you seen physical restraints used with the elderly in their own homes?*						
Yes, n (%)	67 (34.5)	42 (55.3)	27 (21.1)	89 (56.7)	225 (40.5)	< .001
No, n (%)	127 (65.5)	34 (44.7)	101 (78.9)	68 (43.3)	330 (59.5)	
*Have you advised the use of physical restraints or used physical restraints in elders’ homes?*						
Yes, n (%)	21 (11.2)	25 (32.1)	13 (10.1)	33 (21.3)	92 (16.7)	< .001
No, n (%)	167 (88.8)	53 (67.9)	116 (89.9)	122 (78.7)	458 (83.3)	
*Have you attended any education classes on physical restraints?*						
Yes, n (%)	46 (24.5)	19 (24.4)	13 (10.4)	73 (50.3)	151 (28.2)	< .001
No, n (%)	142 (75.5)	59 (75.6)	112 (89.6)	72 (49.7)	385 (71.8)	

### Participation in education classes about physical restraints

Among 475 family caregivers and 536 home care providers (response rate: 50.5%) who answered the question about participation in physical restraint education classes, the proportion of those who had participated once or more (the “once” and “twice or more” options were combined because the proportion of participants who answered as such was small) was significantly higher among home care providers (28.2%) than family caregivers (3.2%; p < .001). Furthermore, the proportion differed significantly among the home care providers (Table 4), with the proportion among care managers about 5 times as high as that among physicians. For all participants, the number of recognized prohibited physical restraint procedures and that of recognized harmful effects were significantly higher, and the average importance rating of reasons for requiring physical restraints was significantly lower among participants who had attended classes than those who had not (all p < .001; Table 5). These same differences were observed among family caregivers alone and home care providers alone (p < .001 to p = .002; Table 5).

**Table 5:** Relationship between participation in education classes and knowledge, recognition, and perceptions

	**Mean ± SD (Median) (95% Confidence Interval of Mean)**		
	**Number of physical restraint procedures recognized as prohibited**	**Number of harmful effects recognized**	**Average importance rating of reasons for requiring physical restraints**
*Have you attended any education classes on physical restraints? (for all participants,* i.e.*, family caregivers and home care providers)*			
Yes (n = 166)	8.16 ± 3.02 (9) (7.68-8.63)	8.65 ± 2.01 (10) (8.34-8.97)	2.53 ± 0.77 (2.47) (2.41-2.66)
No (n = 845)	4.70 ± 3.34 (4) (4.47-4.93)	6.73 ± 3.04 (7) (6.52-6.93)	3.03 ± 0.76 (3.00) (2.98-3.09)
p-value	< .001	< .001	< .001
*Have you attended any education classes on physical restraints? (for family caregivers)*			
Yes (n = 15)	7.08 ± 3.32 (7) (4.98-9.19)	8.57 ± 2.21 (10) (7.30-9.85)	2.36 ± 0.98 (2.41) (1.74-2.99)
No (n = 460)	3.75 ± 3.25 (3) (3.44-4.06)	5.87 ± 3.24 (6) (5.56-6.17)	3.25 ± 0.75 (3.25) (3.17-3.32)
p-value	.002	.001	.001
*Have you attended any education classes on physical restraints? (for home care providers)*			
Yes (n = 151)	8.25 ± 2.99 (9) (7.76-8.74)	8.66 ± 2.00 (10) (8.34-8.99)	2.54 ± 0.76 (2.47) (2.42-2.67)
No (n = 385)	5.80 ± 3.10 (6) (5.48-6.12)	7.74 ± 2.42 (8) (7.49-7.98)	2.79 ± 0.70 (2.81) (2.72-2.87)
p-value	< .001	< .001	.001

SD = Standard Deviation.

## Discussion

In this study, family caregivers were aware of significantly fewer recognized prohibited physical restraint procedures and recognized harmful effects of physical restraint than home care providers, and differences among home care providers were also significant. Similarly, the importance rating of reasons for requiring physical restraints was significantly higher among family caregivers than home care providers and differed significantly among the home care providers. Moreover, these differences were related to participation in physical restraint education classes. While more than a few family caregivers had wavered over using physical restraints, a substantial proportion of home care providers had seen them used in elders’ homes.

### Knowledge and perceptions of physical restraints

The difference in knowledge of physical restraint procedures found between family caregivers and home care providers in this study agrees with an earlier report showing that most family caregivers misidentified several physical restraint procedures as not involving physical restraint use [5]. The difference in knowledge of the harmful effects of physical restraints between family caregivers and home care providers is also consistent with earlier reports showing family members’ positive perceptions of physical restraints [9]. In the present study, for example, compared to home care providers, family caregivers were more likely to perceive physical restraints as important for securing the safety of frail elders. These positive attitudes may be associated with the prevalence of physical restraint use, as they are with formal caregivers and in other countries [30,31]. As such, family caregivers might use physical restraints with home-dwelling elders more readily than formal caregivers would do with institutionalized elders; for example, with the intention of securing their safety.

In this study, we observed significant differences among home care providers (i.e., home helpers, visiting nurses, visiting physicians, and care managers) in their knowledge of physical restraint procedures and knowledge of harmful effects of physical restraints: knowledge of both was highest among care managers and lowest among physicians (with nurses the next lowest). As for the importance ratings given to reasons for requiring physical restraints, physicians and nurses justified use significantly more often than did care managers, who justified use the least. One possible reason is that, compared to other home care providers, care managers more frequently participate in physical restraint education classes. In Japan, physicians and nurses experience physical restraints being used in hospitals as an unavoidable method of treating patients safely [32,33]. Health professionals such as physicians and nurses in other countries too might also assess physical restraints positively [2,34,35] and physical restraint use was part of nurses’ professional training and education, at least in the past.

### The need to survey family caregivers’ use of physical restraints

In this study, 40.5% of home care providers had observed physical restraints used in elders’ homes, suggesting that family caregivers’ use is not so rare. In addition, 20.1% of family caregivers reported wavering over such use. We speculate that had home care providers known all 11 prohibited physical restraint procedures, they would have noticed even more physical restraints during their home visits. Similarly, had family caregivers known more about physical restraint procedures, many more family caregivers would have answered that they had wavered over their use at home. Therefore, the prevalence of physical restraints used by family caregivers might well be high enough to warrant a sensitive survey of their use in order to develop effective and feasible interventions.

Physical restraints are potentially abusive because their essential feature is to restrict a person’s activities, and they can become even more abusive when family caregivers heedlessly persist in using them because, as we observed in this study, family caregivers are less aware than home care providers of the harmful effects of physical restraints. One potentially useful approach to this problem is to focus on why family caregivers use physical restraints at home. For example, awareness of the harmful effects of physical restraints could be one factor that can help family caregivers to distinguish abusive from non-abusive physical restraints.

### Support for family caregivers

This study showed that 96.8% of family caregivers had never participated in physical restraint education classes; thus, most had never had an opportunity to learn about prohibited physical restraints. In fact, the subject of physical restraints is rarely discussed in classes for family caregivers in Japan. Uninformed family caregivers might continue using physical restraints without noticing their harmful effects, including progressive frailty induced by the use of physical restraints, which is easily mistaken for the natural course of aging, and fall-related injuries associated with physical restraints because of the ineffectiveness of restraints in preventing falls [36]. Furthermore, family caregivers can hardly be expected to distinguish between all abusive physical restraints and necessary physical restraints (i.e., physical restraints used to secure the safety of frail elders) [37] because necessary physical restraints can evolve into abusive physical restraints, as shown in the continuous spectrum of abusive behavior [38]. Because family caregivers need both knowledge and skills to provide adequate care [39-41], home care providers must themselves have an appropriate understanding of physical restraints so that they can advise family caregivers not to use abusive ones. Multicomponent intervention programs [42], including fundamental education and instruction by home care providers to improve a family’s knowledge and perceptions of physical restraints (shown in this study to be poor) may reduce unnecessary and abusive physical restraint use.

In this study, knowledge and perceptions of physical restraints differed significantly among home care providers, which suggests the need to standardize their knowledge of physical restraints to ensure that family caregivers receive consistent advice. In particular, a prominent difference existed between care managers and visiting physicians, who were the top 2 home care providers that family caregivers would consult when uncertain about using physical restraints themselves. In fact, physical restraints are still used by staff in elder care facilities where a lack of information about alternatives to physical restraints has been reported [30], and even less information is available to guide the use of physical restraints in elders’ homes [2]. Educational interventions designed to improve a family’s knowledge and perception of physical restraints might also contribute to reduced use of unnecessary physical restraints with institutionalized elders, given that families tend to believe physical restraints can guarantee their security and safety, with some family members even favoring their use [2,9].

Although the use of certain temporary physical restraints by family caregivers might be acceptable [26,37], others might violate human rights, and it is estimated that family caregiving for home-dwelling elders will only increase worldwide as aging populations continue to grow. As yet, there are no evidence-based interventions to reduce elder abuse by family caregivers [43]. The organization and collaboration of home care providers needed to support family caregivers to prevent such abuse is therefore an international issue and a future challenge that most countries, including Japan, must face [37,44-46].

### Limitations

The present study has several limitations. First, our findings might reflect the unique characteristics of Japan and those of specific regions of Japan. For example, nursing staffs’ attitudes toward physical restraint use with nursing home residents have been shown to differ among European countries [8], and cultural differences in health care systems may produce different perceptions and attitudes toward such use in elders’ homes. Even so, this study can still offer lessons and suggestions for other nations given that Japan has the world’s oldest population and that most caregiving is performed by family caregivers even after the introduction of the LTCI system [11]. In addition, the study could conceivably reflect a substantial part of life in Japan because LTCI is available across the country and the proportion of people aged ≥65 years in the areas studied almost equals that in Japan as a whole. Second, this study utilized those physical restraint procedures and harmful effects of physical restraints published by the Ministry of Health, Labour and Welfare of Japan. However, physical restraint procedures have been variously defined among studies [47] and the list of harmful effects is incomplete. As a result, chemical restraints were included in the list of physical restraint procedures, while the list of harmful effects did not include decrease in activities of daily living or exacerbation of behavioral and psychological symptoms of dementia. Therefore, this study did not examine every harmful effect of physical restraints. At the very least, however, these lists have been generally used in the field of elderly care in Japan, and this study showed that differences in knowledge of physical restraints exist between family caregivers and home care providers and among home care providers themselves. Third, the response rate varied among the different groups of home care providers, which could have influenced the results. The response rate of care managers was the highest, possibly reflecting their having the highest degree of knowledge of physical restraints. Fourth, this study did not aim to assess the prevalence of physical restraint use in home-dwelling elders. Nevertheless, our results indicate that the use of physical restraints is commonplace among family caregivers: 20.1% of family caregivers reported having wavered over using them and 40.5% of home care providers had seen them used in homes. It would appear then that a more detailed survey on physical restraint incidents by family caregivers is necessary.

## Conclusions

This study demonstrated that the degree of knowledge and perceptions of physical restraint use with home-dwelling elders differed between family caregivers and home care providers and were also diverse among home care providers. Home care providers should acquire standardized and appropriate knowledge and perceptions of physical restraints in order to help family caregivers minimize unnecessary and abusive physical restraint use with elders living at home. The results also suggested that the prevalence of physical restraint use by family caregivers may be high enough to warrant a survey, the results of which would contribute to developing effective and feasible interventions. Elucidating the determinants behind family caregivers’ physical restraint use is needed to minimize non-temporary, non-emergency, and substitutable physical restraints. As a next step, we need to conduct qualitative studies investigating potential determinants and decision-making processes in physical restraint use by family caregivers, followed by quantitative studies of the prevalence of such use with home-dwelling elders.

## Abbreviation

LTCI: Long-term care insurance.

## Competing interests

The authors declare that they have no competing interests.

## Authors’ contributions

SK designed and obtained funding for this study. SK and TO acquired and analyzed the data. SK prepared the first draft of this manuscript. TO provided critical revisions. Both authors read and approved the final manuscript.

## Pre-publication history

The pre-publication history for this paper can be accessed here:

http://www.biomedcentral.com/1471-2318/14/39/prepub

## Supplementary Material

Additional file 1 Proportions of participants who knew each of the 11 prohibited physical restraint procedures and those who knew each of the 10 harmful effects of physical restraints, and the importance rating for each of the 17 reasons for requiring physical restraint use. Click here for file
